# The Effect of Skeletal Muscle-Specific Creatine Treatment on ALS NMJ Integrity and Function

**DOI:** 10.3390/ijms241713519

**Published:** 2023-08-31

**Authors:** Agnes Badu-Mensah, Xiufang Guo, Roxana Mendez, Hemant Parsaud, James J. Hickman

**Affiliations:** 1 NanoScience Technology Center, University of Central Florida, 12424 Research Parkway, Suite 400, Orlando, FL 32826, USA; agnes.badumensah@gmail.com (A.B.-M.); xiufang.guo@ucf.edu (X.G.); mendezr@knights.ucf.edu (R.M.); hparsaud@knights.ucf.edu (H.P.); 2 Burnett School of Biomedical Sciences, College of Medicine, University of Central Florida, Orlando, FL 32816, USA

**Keywords:** ALS, skeletal muscle, creatine, BioMEMs, SOD1, iPSCs, microfluidic, NMJ

## Abstract

Although skeletal muscle (hSKM) has been proven to be actively involved in Amyotrophic Lateral Sclerosis (ALS) neuromuscular junction (NMJ) dysfunction, it is rarely considered as a pharmacological target in preclinical drug discovery. This project investigated how improving ALS hSKM viability and function effects NMJ integrity. Phenotypic ALS NMJ human-on-a-chip models developed from patient-derived induced pluripotent stem cells (iPSCs) were used to study the effect of hSKM-specific creatine treatment on clinically relevant functional ALS NMJ parameters, such as NMJ numbers, fidelity, stability, and fatigue index. Results indicated comparatively enhanced NMJ numbers, fidelity, and stability, as well as reduced fatigue index, across all hSKM-specific creatine-treated systems. Immunocytochemical analysis of the NMJs also revealed improved post-synaptic nicotinic Acetylcholine receptor (AChR) clustering and cluster size in systems supplemented with creatine relative to the un-dosed control. This work strongly suggests hSKM as a therapeutic target in ALS drug discovery. It also demonstrates the need to consider all tissues involved in multi-systemic diseases, such as ALS, in drug discovery efforts. Finally, this work further establishes the BioMEMs NMJ platform as an effective means of performing mutation-specific drug screening, which is a step towards personalized medicine for rare diseases.

## 1. Introduction

Amyotrophic Lateral Sclerosis (ALS), or Lou Gehrig’s disease, is a multi-systemic, adult-onset disease that leads to death within 2–5 years of diagnosis [1]. It is a neurogenerative disease with about 5000 people diagnosed annually in the United States. Although the phenotype is similar between patients in many regards, ALS cases have been categorized into two groups: familial ALS (fALS) and sporadic ALS (sALS). FALS cases consist of about 10% of all known ALS cases and are associated with earlier disease onset, while the remaining 90% of patients fall into the sALS category [1]. While the etiology of the disease is not fully elucidated, dysfunction in many tissue types, including motoneurons, skeletal muscle, astrocytes, microglia, and Schwann cells, has been implicated in ALS pathology [1,2]. Currently, mutations in over 50 genes have been associated with ALS. However, most patients with known ALS genetic backgrounds have mutations in the chromosome 9 open reading frame 72 (*C9orf72*), Fused Sarcoma (FUS), Tar-DNA binding protein 43 (*TDP-43*), or Superoxide dismutase 1 (*SOD1*) genes [3].

There is currently no cure for ALS despite decades of research. The existing FDA approved drugs, Riluzole and Edaravone, only marginally improve the life expectancy of patients [4]. Thus, there is a critical need for more effective therapeutic development approaches and methods. However, the lack of disease models with translational capability that can recapitulate patient outcomes is one of the main confounding challenges in ALS drug discovery. Although transgenic animal models have been instrumental in improving the field’s general understanding of the disease, they generally fail to predict clinical efficacy and safety outcomes of therapeutics in patients, thus, numerous ALS therapeutics have failed in clinical trials [5]. Another challenge has been a lack of consensus on the pathogenesis of ALS. Some scientists purport ALS progression to be a neuronal dying-forward mechanism while others claim a dying-back mechanism. However, recent findings have both groups agreeing that neuromuscular junction (NMJ) disruption is one of the earliest indications of ALS pathology [6]. As much as this finding provides researchers a common site to investigate disease progression, little is known about the cause of NMJ dysfunction; therefore, investigating factors involved in ALS NMJ dysfunction in human models is necessary.

It has been reported that protein aggregates, down-regulation of transport proteins, and mitochondrial dysfunction together result in impaired axonal transport, which contributes to NMJ dysfunction by disrupting presynaptic action potential propagation [7,8]. Other studies have demonstrated intrinsic morphological and functional deficits in other tissues involved in the tripartite synapse that could have a direct impact on NMJ formation, maintenance, and function [6,9,10]. However, there is still doubt concerning the involvement of skeletal muscle or Schwann cells in ALS NMJ dismantling [6]. This has led to the development of ALS patient-derived NMJ models to delineate the contribution of ALS hSKM and hMN to NMJ dysfunction [11,12]. While results indicated significantly reduced NMJ formation, NMJ stability, and contraction fidelity, as well as increased fatigue index in co-cultures containing either ALS hSKM or hMN, observed functional defects in systems with hSKM were relatively more severe, thus demonstrating the importance of the hSKM’s role in ALS NMJ integrity and function [11]. A consequential question addressed in this report was whether specific therapeutic targeting of the ALS hSKM confers any benefit on human ALS NMJ function.

Based on preliminary hSKM-only experimentation, creatine was selected among four drugs for subsequent NMJ experiments based on its demonstrated ability to improve ALS myoblast fusion and mitochondrial function. NMJ systems with creatine dosing specifically on the hSKM side were investigated with the aim of evaluating the effect of ALS hSKM improvement on ALS NMJ integrity and function. Skeletal muscle-specific creatine treatment demonstrated comparatively increased NMJ numbers, contraction fidelity, and stability. Also, there was an observed decrease in NMJ fatigue indices across all treated ALS NMJ systems. Morphological assessment of these creatine-treated ALS NMJs indicated a significant increase in acetylcholine receptor cluster number per myotube as well as size of the clusters. Findings from this study buttress the relevance of considering ALS SKM as an active contributor to ALS NMJ dysfunction. The study also confirms the skeletal muscle as a therapeutic target in ALS, using a number of clinically relevant functional parameters. Lastly, it demonstrates that this human-based BioMEMs platform is suitable for investigating multi-faceted diseases like ALS and for preclinical drug efficacy and toxicity screening.

## 2. Results

### 2.1. Effect of Therapeutics on ALS Myotubes

**Figure 1:**
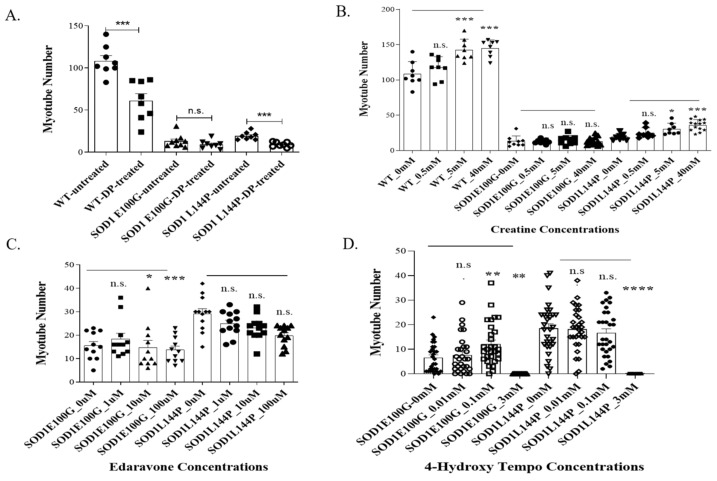
Quantification of the number of myotubes formed in WT and SOD1 muscle cultures after treatment with different chemicals or formulations. (**A**) Deanna Protocol (DP), (**B**) Creatine, (**C**) Edaravone, (**D**) 4-Hydroxy Tempo (Tempol). Data are presented as the mean number of myotubes per 0.137 µm. Error bars: SEM * *p* < 0.1, ** *p* < 0.01, *** *p* < 0.001, **** *p* < 0.0001, n.s. = not significant.

**Figure 2:**
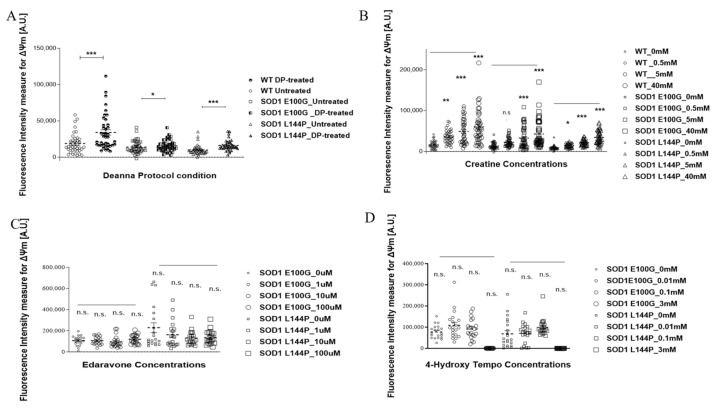
Scatter plots of the average inner mitochondrial membrane potential by TMRE measurement of SOD1 and WT hSKM after treatment with (**A**) Deanna Protocol (DP), (**B**) Creatine, (**C**) Edaravone, and (**D**) 4-Hydroxy Tempo (Tempol). TMRE signal was measured in and reported for fluorescent intensity. Error bars: SEM * *p* < 0.1, ** *p* < 0.01, *** *p* < 0.001, n.s. = not significant.

It is increasingly acknowledged that ALS-associated mutations negatively impact hSKM morphology, metabolism, and function. Reduced fusibility, lower contractile force output, and decreased mitochondrial function are among commonly reported deficits across many different ALS mutations. Interestingly, these defects are mostly linked to oxidative stress [13,14,15,16]. However, there is limited knowledge about how existing ALS therapeutics help mitigate the negative effect of reactive oxygen species (ROS) on the diseased muscle [17,18] and how the drugs targeting ROS can improve the physiology of ALS muscle. Thus, four potential therapeutics were tested in our established ALS-NMJ system [11,12] based on their known mechanisms of action and our experience: (A) ROS scavengers, edaravone, an FDA-approved ALS drug; (B) 4-hydroxy tempo (Tempol), is a pleotropic antioxidant whose protective effect has been observed in skeletal muscle and other tissues through a mechanism related to mitochondria dysfunction [19]; (C) The Deanna Protocol (DP), a holistic ALS treatment which has demonstrated beneficial effect on ALS-motoneurons [20] and ALS-NMJ [12]; (D) Creatine (Cr), which has been shown to participate in ATP recycling and is well-known for its effect on muscle building through multiple mechanisms [12,19,20,21,22]. Among the treated cultures, only those dosed with creatine demonstrated both enhanced fusion and mitochondrial function (Figure 1). There was a general dose-dependent increase in the number of myotubes across all mutations in creatine-treated cultures compared to untreated controls (Figure 1B). Conversely, inner mitochondrial membrane potential (Δψ_m_) was significantly improved in both DP- and creatine-treated cultures, while edaravone- and Tempol-treated cultures had decreased Δψ_m_ (Figure 2).

Based on the above outcomes, creatine was chosen for subsequent NMJ experiments. Creatine experiments were conducted with the dosage of 5 mM as it was the lowest concentration to show significant changes in the number of myotubes and mitochondrial function. Moreover, there was generally no significant difference between 5 mM and 40 mM outcomes across the parameters measured.

### 2.2. Effect of ALS hSKM-Specific Creatine Treatment on NMJ Numbers

**Figure 3:**
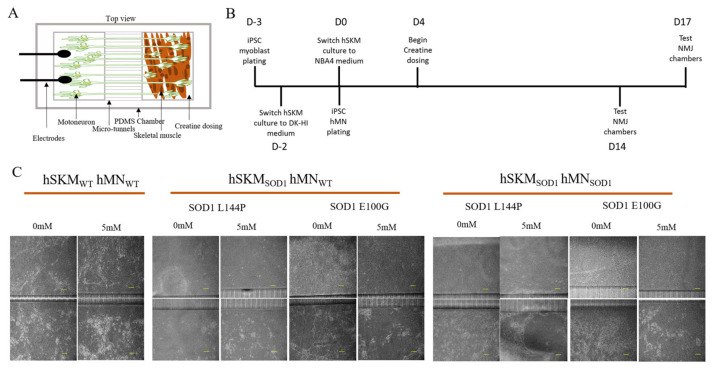
Illustration of SOD1 and WT NMJ culture with and without creatine treatment. (**A**) Cartoon illustration of the NMJ chamber. (**B**) Experimental timeline for iPSC hSKM and hMN plating and subsequent creatine treatment. (**C**) Phase images for the co-cultures of hSKM_WT_ hMN_WT_, hSKM_SOD1_ hMN_WT_ and hSKM_SOD1_ hMN_SOD1_ with/without creatine. Scale bar = 100 µm.

**Figure 4:**
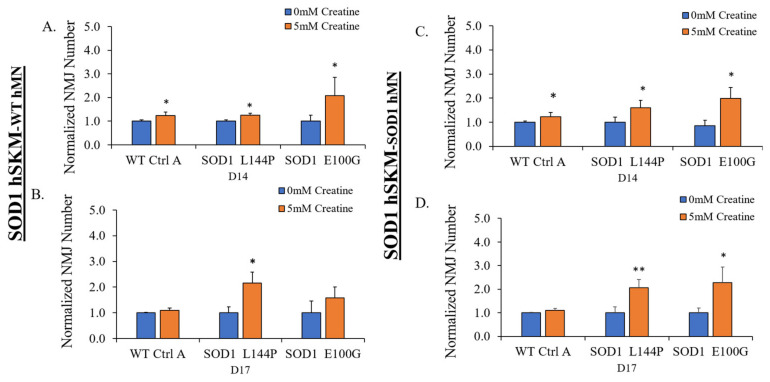
Quantification of NMJ numbers in NMJ cultures with and without creatine treatment at D14 and D17 days of differentiation. The results from different co-cultures are illustrated. (**A**) D14 hSKM_SOD1_ hMN_WT_, (**B**) D17 hSKM_SOD1_ hMN_WT_, (**C**) D14 hSKM_SOD1_ hMN_SOD1_, and (**D**) D17 hSKM_SOD1_ hMN_SOD1_. Result: Mean. Error bars: SEM * *p* < 0.1, ** *p* < 0.01.

Preliminary hSKM-only experiments demonstrated significantly increased ALS myoblast fusibility, which improved the number of myotubes in creatine-treated cultures. The number of NMJs was quantified as an estimate of NMJ formation with or without creatine supplementation on days 14 and 17 of co-culture (Figure 3). SOD1^L144P^ and SOD1^E100G^ systems that received hSKM-specific creatine treatment had higher NMJ numbers relative to untreated groups on days 14 and 17 of co-culture (Figure 4). This observation was true for both hSKM_SOD1_-hMN_WT_ and hSKM_SOD1_-hMN_SOD1_ co-cultures. Interestingly, increased NMJ numbers were also noted in hSKM_WT_-hMN_WT_ systems treated with creatine. Collectively, these results indicate that muscle-specific creatine treatment enhances NMJ formation in ALS cultures.

### 2.3. Effect of ALS hSKM-Specific Creatine Treatment on NMJ Fidelity

**Figure 5:**
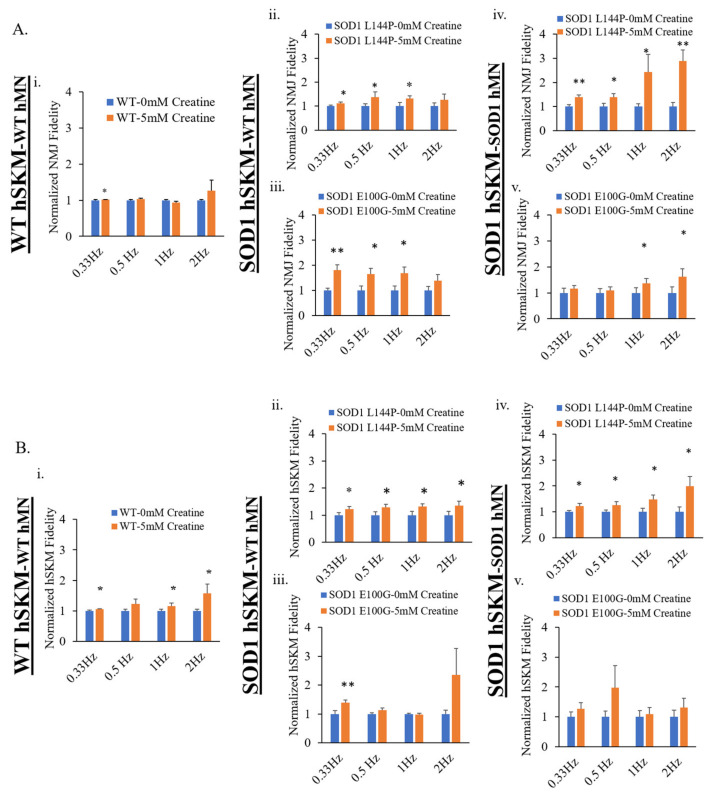
Quantification of NMJ fidelity and direct muscle contraction fidelity in the D14 NMJ co-cultures with and without creatine treatment. (**A**) NMJ fidelity collected under MN stimulation. (**B**) SKM contraction fidelity collected under SKM direct stimulation. Results from the NMJ co-cultures of different genetic combinations are displayed: (**i**) WT, (**ii**) SOD1^L144P^ SKM-WT MN, (**iii**) SOD1^E100G^ SKM-WT MN, (**iv**) SOD1^L144P^ SKM-SOD1^L144P^ MN, (**v**) SOD1^E100G^ SKM-SOD1^E100G^ MN. Data: Mean, Error bars: SEM * *p* < 0.1, ** *p* < 0.01.

**Figure 6:**
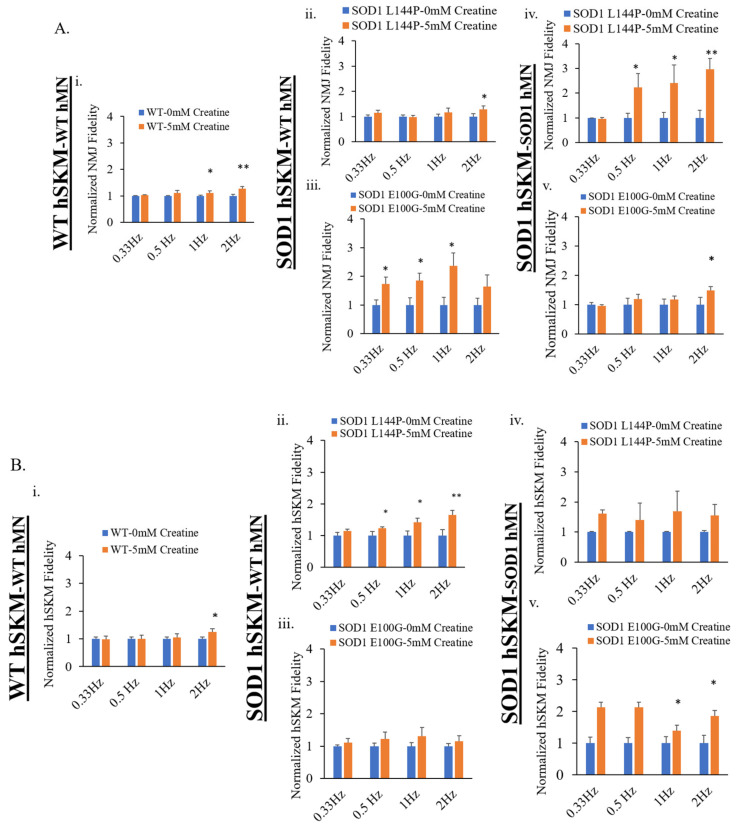
Quantification of NMJ fidelity and direct muscle contraction fidelity in the D17 NMJ co-culture with and without creatine treatment. (**A**) NMJ fidelity collected under MN stimulation. (**B**) SKM contraction fidelity collected under SKM direct stimulation. Results from the NMJ co-cultures of different genetic combinations were displayed: (**i**) WT, (**ii**) SOD1^L144P^ SKM-WT MN, (**iii**) SOD1^E100G^ SKM-WT MN, (**iv**) SOD1^L144P^ SKM-SOD1^L144P^ MN, (**v**) SOD1^E100G^ SKM-SOD1^E100G^ MN. Data: Mean. Error bars: SEM, * *p* < 0.1, ** *p* < 0.01.

The next clinically relevant parameter evaluated was NMJ fidelity. This is a measure of the reliability of motoneuron-to-skeletal muscle action potential transmission and its resultant contraction at increasing frequencies [12]. One innervated myofiber from each NMJ chamber system was randomly selected for recording after stimulation of MNs at frequencies ranging from 0.33 to 2 Hz at 5 V for 15 s. Compared to untreated, hSKM-specific creatine-treated systems of both hSKM_SOD1_-hMN_WT_ and hSKM_SOD1_-hMN_SOD1_ had increased NMJ fidelity on both days 14 and 17, especially at higher frequencies (i.e., 1 and 2 Hz) (Figure 5). Similarly, ALS hSKM in the treated cultures had notably higher contraction fidelity upon direct muscle stimulation compared to untreated (Figure 6). Together, these results demonstrate that hSKM-specific creatine treatment improve ALS NMJ muscle contraction fidelity.

### 2.4. Effect of ALS hSKM-Specific Creatine Treatment on NMJ Stability

**Figure 7:**
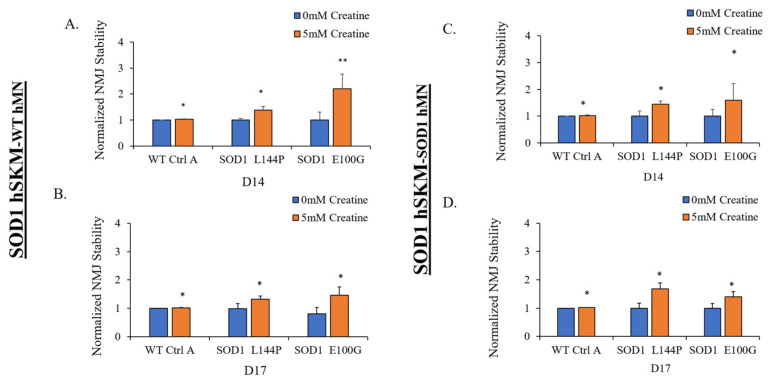
Quantification of NMJ stability with and without creatine treatment tested at D14 and D17 of the NMJ culture utilizing different genetic combinations. (**A**,**B**) hSKM_SOD1_ hMN_WT_ tested at D14 (**A**) and D17 (**B**). (**C**,**D**) hSKM_SOD1_ hMN_SOD1_, tested at D14 (**C**) and D17 (**D**). Data: Mean. Error bars: SEM * *p* < 0.1, ** *p* < 0.01.

The therapeutic effect of creatine on NMJ stability was also assessed. This is because a decline in NMJ stability is a commonly reported ALS clinical symptom [23]. This parameter was measured to evaluate preservation of NMJ function in ALS co-cultures following exposure to a paradigm of NMJ stimulation at increasing frequency, as in fidelity testing. NMJ stability was calculated as the number of NMJs counted after the fidelity testing paradigm divided by number of NMJs pre-fidelity testing [9,12]. Compared to the untreated condition, NMJs in the creatine-treated ALS co-cultures were found to be more stable (Figure 7). The observation was made in hSKM_SOD1_-hMN_WT_ and hSKM_SOD1_-hMN_SOD1_ on both day 14 and 17 of co-culture. These results demonstrate that hSKM-specific creatine treatment increases ALS NMJ stability.

### 2.5. Effect of ALS hSKM-Specific Creatine Treatment on NMJ Fatigability

**Figure 8:**
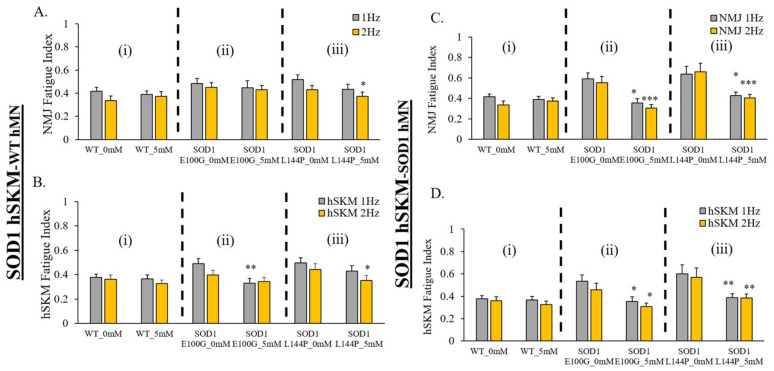
Quantification of the fatigue index for NMJ-stimulated SKM and direct SKM stimulation in the D14 NMJ co-culture with and without creatine treatment. (**A**) NMJ fatigue index collected under MN stimulation. (**B**) SKM fatigue index collected under SKM direct stimulation. Results from the NMJ co-cultures of different genetic combinations are displayed: (**A**,**B**) In the co-culture of hSKM_SOD1_ hMN_WT_, fatigue index of NMJ (**A**) and SKM (**B**). (**C**,**D**) In the co-culture of hSKM_SOD1_ hMN_SOD1_, fatigue index of NMJ (**C**) and SKM (**D**). (**i**) WT (**ii**) SOD1 E100G (**iii**) SOD1 L144P. Data: Mean. Error bars: SEM * *p* < 0.1, ** *p* < 0.01, *** *p* < 0.001.

**Figure 9:**
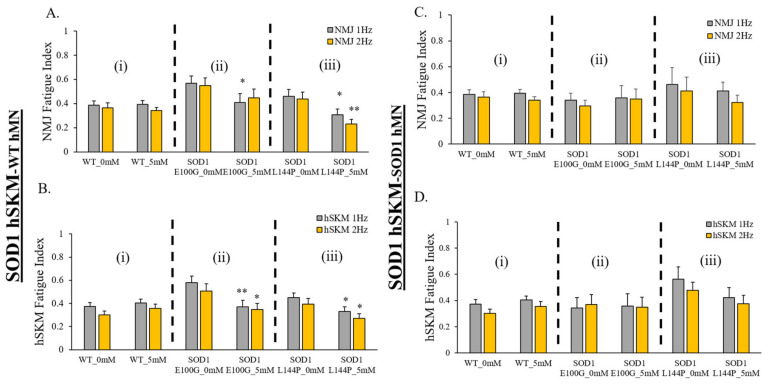
Quantification of the fatigue index for NMJ-stimulated SKM and direct SKM stimulation in the D17 NMJ co-culture with and without creatine treatment. (**A**) NMJ fatigue index collected under MN stimulation. (**B**) SKM fatigue index collected under SKM direct stimulation. Results from the NMJ co-cultures of different genetic combinations are displayed: (**A**,**B**) In the co-culture of hSKM_SOD1_ hMN_WT_, fatigue index of NMJ (**A**) and SKM (**B**). (**C**,**D**) In the co-culture of hSKM_SOD1_ hMN_SOD1_, fatigue index of NMJ (**C**) and SKM (**D**). (**i**) WT (**ii**) SOD1^E100G^ (**iii**) SOD1^L144P^. Error bars: SEM * *p* < 0.1, ** *p* < 0.01.

The last functional parameter assessed was NMJ fatigue. Increased fatigue and decreased mechanical output are clinical phenotypes reported among ALS patients [24]. Increased fatigue has been linked to poor patient prognosis and survivability [25,26]. Thus, the therapeutic effect of creatine on NMJ and hSKM fatigue was evaluated in our systems by measuring NMJ and hSKM fatigue indices at relatively higher frequencies of stimulation (i.e., 1 Hz and 2 Hz). Results demonstrated relatively decreased NMJ fatigue in creatine-treated systems on days 14 (Figure 8) and 17 of co-culture (Figure 9), except in hSKM_SOD1 E100G_-hMN_SOD1 E100G_ at day 14. This observation was also true for direct measurement of the ALS hSKM fatigue index (Figure 8 and Figure 9).

### 2.6. Effect of ALS hSKM-Specific Creatine Treatment on AChR Cluster Number per Myotube and Innervated AChR Cluster Size

**Figure 10:**
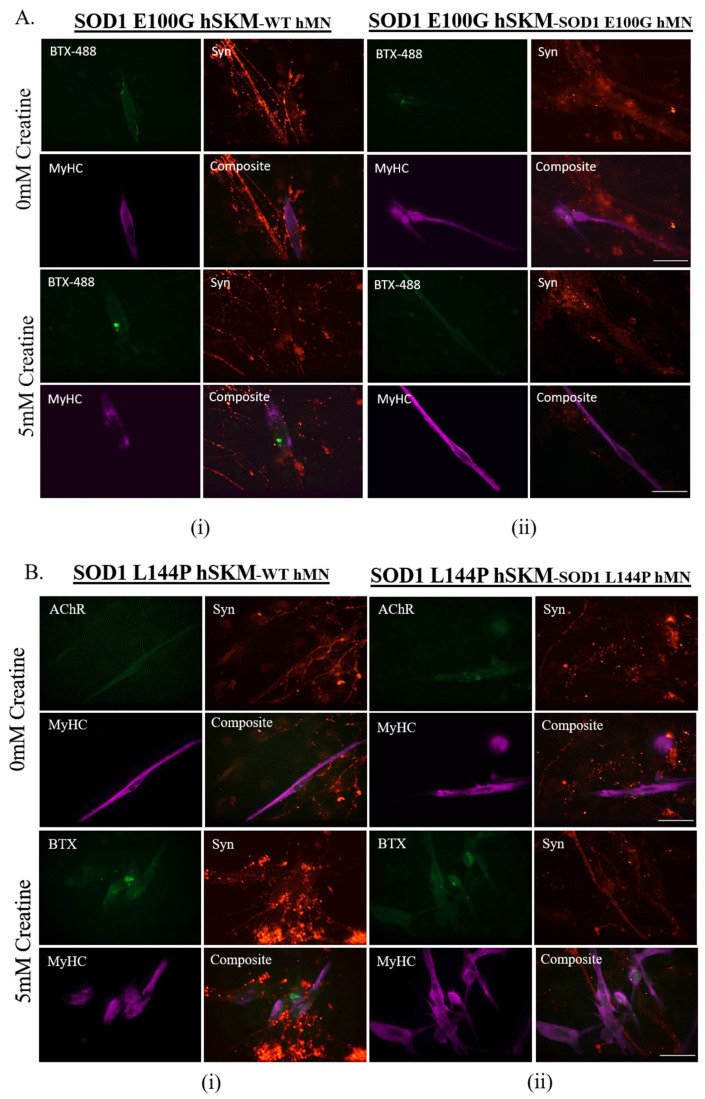
Immunocytochemistry analysis of the D17 NMJ co-culture with and without creatine treatment utilizing the markers BTX-488 (green) for AchR and Myosin Heavy Chain (MyHC) (red) or Synaptophysin (syn, red) for myotubes. (**A**) For the co-cultures containing the SOD1 E100G mutation. (**B**) For the co-cultures containing the SOD1 L144P mutation. Sample images from the NMJ co-cultures of different genetic combinations are displayed: hSKM_SOD1_ hMN_WT_ and hSKM_SOD1_ hMN_SOD1_. Scale bar = 100 µm.

Postsynaptic propagation of action potentials is dependent on the binding of acetylcholine to its receptor on the skeletal muscle membrane. ALS skeletal muscle was previously reported to have reduced AChR clusters, which could potentially affect synaptic transmission [9]. Having observed improved NMJ numbers, stability, fidelity and fatigability, the post-synaptic terminal of the NMJ was assessed to determine whether creatine treatment had any therapeutic impact on NMJ morphology. AChR cluster number and size were quantified in the creatine-treated condition and compared with the untreated control by immunocytochemical staining with myosin heavy chain for myofibers, synaptophysin (Syn) for axonal terminals, and bungarotoxin conjugated to fluorophore 488 (BTX-488) for AchRs (Figure 10). Quantification of the ICC result in Image J revealed a significant increase in AChR cluster size and/or cluster number per myotube in the treated condition compared to untreated, indicating enhanced post-synaptic AChR clustering with creatine treatment.

## 3. Discussion

The objective of this study was to evaluate the therapeutic effect of muscle-specific creatine treatment on ALS NMJ integrity and function. In addition to its general function in locomotion, posture, breathing and gait, the skeletal muscle (hSKM) is a known major metabolic regulator and is partly responsible for whole body homeostasis [27]. As such, any impairment to this organ has detrimental effects clinically. In many disease states, poor prognosis has been linked to excessive skeletal muscle wasting, and in ALS, rapid weight loss has been linked to shorter survival times [28]. Impaired hSKM metabolism, morphology, and function have been reported to precede disease onset and to contribute to NMJ dysfunction [29,30,31,32]. However, it is often overlooked and rarely considered in ALS pre-clinical drug discovery efforts. This study explored whether treatments targeting improved ALS skeletal muscle function confers any benefit to the NMJ.

**Figure 11:**
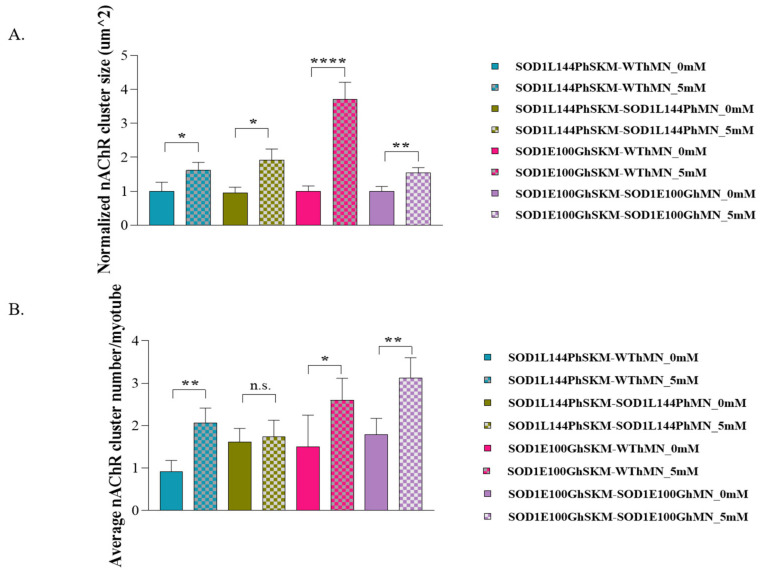
Quantification of nAChR clusters in the NMJ culture. (**A**) nAchR cluster size (**B**) nAchR cluster number per myotube in the co-cultures of hSKM_SOD1_ hMN_WT_ and hSKM_SOD1_ hMN_SOD1_ with/without creatine treatment at days 17 of differentiation. Data: Mean. Error bars: SEM * *p* < 0.1, ** *p* < 0.01, **** *p* < 0.0001, n.s. = not significant.

The study was carried out in microfluidic NMJ systems developed from ALS patient-derived iPSCs, which had been previously characterized to have reduced NMJ formation propensity, decreased contraction synchrony, and diminished stability, as well as increased fatigability compared to healthy controls [12]. Based on the drug testing results from the hSKM-only cultures demonstrating enhancement in both number of myotubes and mitochondrial function by creatine treatment in ALS and WT hSKM cultures, creatine was selected to be used for subsequent NMJ experiments (Figure 1 and Figure 2), and the result clearly indicated the beneficial effect of creatine on ALS-NMJ (Figure 4, Figure 5, Figure 6, Figure 7, Figure 8, Figure 9, Figure 10 and Figure 11). Creatine is reported to increase energy availability, lean muscle mass, muscular strength, overall protein expression, myogenic differentiation, intracellular Ca^2+^ handling, and survival in dystrophic skeletal muscle cells in addition to inducing antioxidative defense in myotube cultures [33,34,35,36]. Moreover, creatine is an over-the-counter supplement that has already been widely used by athletes for muscle building. Thus, findings regarding its effect in ALS could lead to immediate application in ALS patients since there are no concerns about its bioavailability, accessibility, and side effects.

The first parameter evaluated was NMJ formation as NMJ regeneration and maintenance are known to be impaired in ALS [6]. hSKM-specific creatine treatment significantly increased NMJ numbers in both SOD1^L144P^ and SOD1^E100G^ co-cultures for both hSKM_SOD1_-hMN_WT_ and hSKM_SOD1_-hMN_SOD1_ compared to untreated controls (Figure 4). This was true on both days 14 and 17 of co-culture. An increase in NMJ numbers in hSKM_SOD1_-hMN_WT_ systems was within expectation since the hSKM side of the chambers were treated (Figure 4A,B). It is worth noting that significantly increased NMJ numbers were also observed in creatine-treated hSKM_SOD1_-hMN_SOD1_ co-cultures. Immunocytochemical assessment of NMJs from treated and untreated conditions post-test provided insight into these observations. While there were no apparent changes to the hMN axons between treated versus untreated in any of the ALS co-culture conditions, ALS hSKM in creatine-treated chambers had significantly larger AChR clusters compared to untreated (Figure 10 and Figure 11). Additionally, myotubes in the treated conditions had a comparatively higher number of AChR clusters per myotube than untreated (Figure 11B). The formation of larger AChR clusters is likely a result of a generally increased protein expression upon creatine supplementation [34,37]. Increased AChR clusters per myotube in turn enhanced NMJ formation possibilities in the creatine condition. Overall, creatine supplementation boosted NMJ formation in ALS co-cultures.

Other than the increase of NMJ formation, the fidelity of NMJ function is another clinically relevant parameter of interest. As previously described, ALS NMJs demonstrate significantly reduced NMJ fidelity compared to hSKM_WT-_hMN_WT_ –co-cultures [12]. NMJ fidelity increased with hSKM-specific creatine treatment, across all ALS systems on both days 14 and 17 co-cultures relative to untreated (Figure 5A and Figure 6A). Similar direct hSKM fidelity assessments indicated a positive outcome (Figure 5B and Figure 6B), in line with the increase in NMJ fidelity. Based on the analysis reported here and from previous work, it is speculated that at least three mechanisms can potentially contribute to this improvement: (A) the improvement of mitochondria function (Figure 2), (B) the increased AchR cluster size (Figure 11), and (C) the increase of myosin heavy chain expression and contraction force. Enhanced inner mitochondrial membrane potential implies increased electron transport chain activity, which in turn improves ATP generation. Also, Δψ_m_ has a positive effect on mitochondrial calcium handling, which plays a critical role in overall cell metabolism and function [38]. Additionally, increased mitochondrial function coupled with the availability of creatine as an energy buffer improved ALS myotube formation, which is an energy-intensive process [39]. Based on morphological analysis, it is likely that the creatine-enhanced hSKM metabolism improved calcium handling and protein expression [33,35]. Beside the significant increase in ALS hSKM Δψ_m_, other studies have reported that creatine mitigates ROS buildup by preventing mitochondrial permeability transition (MPT) pore formation [40,41]. Specifically, scientists have demonstrated that creatine, once converted to phospho-creatine, intracellularly attenuates mitochondrial membrane uncoupling by directly binding and structurally stabilizing the lipid bilayer [42]. A previously published work from our laboratory demonstrated increased skeletal muscle contraction peak force and myosin heavy chain expression with creatine treatment [34]. These findings together provide the mechanistic cellular basis for the observed improvement in contraction fidelity and fatigue index in the creatine-treated condition (Figure 5 and Figure 6).

Another observation was enhanced NMJ stability in creatine-treated co-cultures relative to the untreated ALS NMJs (Figure 7). This may also be attributed to improved post-synaptic endplate maturation as well as increased energy availability. ALS myotubes have been reported to have reduced AChR clustering, which is critical in NMJ formation, maintenance, and synaptic transmission of action potentials [9,10]. Increased AChR cluster number and size in ALS NMJ co-cultures, in addition to improved mitochondrial function and energy availability, supports the relatively increased NMJ stability in creatine-treated NMJ systems (Figure 10 and Figure 11).

A question still left unanswered is how creatine is associated with AChR expression. While there are no experiments that link creatine supplementation and AChR upregulation to the best of our knowledge, there are reports which indicate that AChR expression and peroxisome proliferator-activated receptor-γ coactivator-1α (PGC-1α) are positively correlated [43]. Also, one rat muscle study showed a marginal increase in PGC-1α mRNA expression upon creatine supplementation [34]. However, a human-based study has reported increased protein and mRNA expression levels of p38 MAPK, an upstream regulator of PGC-1α that is upregulated with creatine supplementation [44,45]. Another in vitro study with rat skeletal muscle demonstrated improved muscle function induced by creatine treatment in which alterations in hypertrophic and mitochondrial biogenesis pathways may have played a role [34]. Thus, this study may be the first to demonstrate an association between AChR expression/clustering and creatine supplementation, but further experimentation may be required to definitively understand this link.

It must be noted that, while there was an overall trend in improvement for contraction fidelity and fatigue index for both NMJ and hSKM upon creatine treatment, this increase was not always statistically significant. Also, NMJ numbers and function in creatine-treated chambers were not totally rescued to levels comparable to healthy conditions. However, this study with creatine demonstrated the critical role of hSKM in NMJ formation and maintenance and suggests that solely focusing on improving one tissue type in a multifactorial disease like ALS may not be an effective approach. Contrarily, targeting a mechanism which is dysfunctional across all tissues involved in ALS pathology, like mitochondrial function, may be promising. Another approach may be the utilization of combination therapies that target more than one common pathway could prove effective. For instance, treatments that concomitantly target ROS reduction and boosting energy levels may be more effective than one that solely targets either pathway.

## 4. Materials and Methods

### 4.1. Tissue Derivation of IPSC-Derived Cell Source

All human hSKM myoblasts and motoneurons used in the study were generated from the iPSC cell lines purchased from the Coriell Institute, Camden, NJ, USA: ND41865 (Wildtype (WT)), ND35662 (SOD1 E100G), ND39032 (SOD1 L144P, recently changed to SOD1 L145F by Coriell). All these cell lines have been de-identified before purchase, and no human subjects were involved in this research. IPSCs from passages 6 to 12 were used in motoneuron and myoblast differentiation. Each cell type was generated using a serum-free small molecule directed protocol and were characterized for their respective markers using immunocytochemistry and flow cytometry. Detailed discussion of the differentiation and characterization of each cell type can be found in previous publications from our laboratory [9,12].

### 4.2. BioMEMs NMJ Chamber Fabrication and Assembly

NMJ chambers were constructed from glass bonded to polydimethylsiloxane (PDMS) casts, as described in our previous publications [12,46]. Briefly, PDMS mold cut-outs were allowed to leach in 70% isopropyl alcohol overnight, after which they were rinsed in sterile water. Chambers were made by firmly pressing down sterilized and dry PDMS cut-outs on plasma-cleaned glass coverslips. The skeletal muscle and motoneuron sides of the chamber were coated with collagen I (ThermoFisher A1048301, Waltham, MA, USA) (100 µL Col I, 4.9 mls 1x PBS, 6 µL acetic acid) and Laminin (ThermoFisher 23017015, Waltham, MA, USA) (3 µg/mL in 1x phosphate buffered saline (PBS)), respectively, and allowed to incubate overnight at 4 °C prior to cell plating. Assembled chambers were brought to room temperature the next day before rinsing either side of each chamber with 1x PBS in preparation for cell plating.

### 4.3. Cell Plating and Maintenance

Plating timelines established for each cell type were based on previous co-culture optimization experiments [12]. The skeletal myoblasts were plated at 400 cells/mm^2^, following thawing and subsequent rinsing of chambers with 1x PBS. These myoblasts were allowed to proliferate in myocult medium (DMEM 1000 mg/L, Stemcell technologies, 36253 plus Myocult-SF supplement, Stemcell technologies 05982, Vancouver, BC, Canada) for one day, after which they were switched to priming medium, DK-HI (DMEM/F12 21041-025 ThermoFisher Scientific, Waltham, MA, USA, 15% vol/vol Knockout Serum Replacement ThermoFisher Scientific 10828-028, 10 ng/mL hepatocyte growth factor (HGF) Peprotech 315-23, 2 ng/mL insulin-like growth factor (IGF), Sigma I1271, 0.1 mM β-mercaptoethanol, ThermoFisher Scientific 31350010, 1% MEM Non-Essential amino acids Solution ThermoFisher Scientific 11140050 and 100 nM dexamethasone, Sigma D4902), for 48 h. Muscle cultures were then switched to differentiation medium (NBActiv4, Brain Bits, Springfield, IL, USA) on the same day hMNs are plated at 1500 cells/mm^2^ on their respective side of the chamber in hMN medium [47]. Skeletal muscle-specific treatment with 5 mM creatine (Sigma Aldrich, C0780, St. Louis, MO, USA) began at day 4 in differentiation and was maintained in muscle cultures until days 14 and 17 of testing. The iPSC hMNs were plated in the laminin-coated side of the chamber, three days after hSKM plating and at day 0 of hSKM differentiation. The hMNs were maintained in hMN medium throughout the culture period, with half medium changes every other day.

### 4.4. ALS Skeletal Muscle Drug and Supplement Treatment

The effect of the Deanna Protocol (DP) (L-arginine (310 × 10^−6^ m), α-ketoglutarate (310 × 10^−6^ m), γ-aminobutyric acid (0.8 × 10^−6^ m), 5-hydroxytryptophan (0.8 × 10^−6^ m), glutamate oxaloacetate transaminase (7100 mU L^−1^) [20], creatine, edaravone (an FDA-approved ALS drug) (Sigma, M70800, St. Louis, MO, USA), and 4-hydroxy tempo (Tempol) (Sigma, 176141) on myoblast fusion and mitochondrial function were studied on ALS hSKM (SOD1 E100G, SOD1 L144P)-only cultures and compared to WT hSKM. Concentration ranges tested for each drug or drug combination were based on previous publications. iPSC hSKM myoblasts were plated on 18 mm plasma-cleaned, collagen-coated glass coverslips and cultured as previously discussed until day 4 of differentiation when drug treatments were initiated. At days 10 and 12 of differentiation, cultures in each treatment condition were assessed for any improvement in myotube formation and mitochondrial function, respectively. Using ImageJ (version 1.8.0), the number of myotubes in each condition was quantified from representative phase-contrast images taken across all cell lines. Aside from the DP cocktail, which had been optimized in previous publications from our laboratory, all other drug candidates were tested within a range of concentrations based on literature reports. Creatine treatments concentrations were 0, 0.5, 5, and 40 mM, edaravone at 0, 1, 10, and 100 µM, while Tempol treatment concentrations were 0, 0.01, 0.1, and 3 mM. Dosing for each drug was done every two days until day 12, when myotubes in both WT and ALS cultures were assayed for mitochondrial function.

### 4.5. NMJ Testing and Quantification

NMJ formation in all co-cultures was tested at days 14 and 17 of muscle differentiation. To determine there was no electrical leakage between the hMN and hSKM sides of each chamber, TEER measurements were taken with EVOM2™ Epithelial Voltohmmeter electrodes (World Precision Instrument, Sarasota, FL, USA). Resistance measurements of 5000 ohms were considered high enough to prevent electrical leakage. NMJ formation was confirmed when skeletal muscle contraction was observed in response to electrical pulsing of the motoneuron chamber. NMJs were selected and assessed for stability, fidelity, and fatigue index after scanning and recording the number of NMJs formed in each chamber. Select NMJs were stimulated for 0.33, 0.5, 1, 2 Hz at 15 s per frequency. After stimulation at increasing frequencies, the number of NMJs were recounted, after which skeletal muscle initially assessed for NMJ testing was directly tested for muscle function. Test recordings were done on an upright phase-contrast microscope (Zeiss Hal 100) connected to a Hamamatsu digital camera (model C8484-05G). NMJ numbers, stability, and fatigue index were measured as previously described by [9,12]. Fatigue index was calculated as 1 − [Area under curve/(Peakforce × Time)].

### 4.6. Immunocytochemistry

NMJ cultures were fixed with 4% paraformaldehyde solution for 15 min following testing and rinsed thrice with 1x PBS. The cultures were permeabilized with 0.1% Triton-X solution for 15 min and blocked with donkey serum blocking buffer (2.5% donkey serum, 1% bovine serum albumin) for one hour. Primary antibodies (i.e., Myosin Heavy Chain (MyHC)) 1:30 (DSHB, A4_1025), Synaptophysin (syn) 1:1000 Fisher Scientific, PA1-1043), were added to the cultures and incubated overnight at 4 °C, after which they were rinsed thrice with 1x PBS. Secondary antibodies were added at 1:250 following the rinses and were incubated at room temperature for two hours. Bungarotoxin-488 (BTX-488) (ThermoFisher, B13422, Waltham, MA, USA) was added in the secondary antibody step at a 1:100 ratio. 4′,6-diamidino-2-phenylindole DAPI Fisher Scientific, D1306 (1:1000) was added during the second rinse of three rinses post-secondary antibody incubation to visualize cell nuclei. Each chamber was imaged inverted and immersed in 1x PBS. Images were taken using a Zeiss Axioscope Spinning disk confocal microscope with the help of Volocity software (version 6.3.0, Perkin Elmer, Waltham, MA, USA).

### 4.7. Mitochondrial Function Assessment

Differentiated myotubes were incubated in 500 nM tetramethylrhodamine (TMRE) solution (Thermofisher Scientific, T669, Waltham, MA, USA) at day 12 of differentiation for 30 min and rinsed with 1x PBS prior to imaging. Fluorescence images of the myotube cultures were taken with a Zeiss spinning disk confocal microscope (Axioskop 2 Mot Plus). The average fluorescence intensity per myotube across all conditions was measured via ImageJ (version 1.8.0), as suggested by Hammond [48].

### 4.8. Acetylcholine Receptor Number and Size Quantification

Chambers used in the assessment were fixed for immunostaining at day 17 of co-culture. The cultures stained for synaptophysin, MyHC, bungarotoxin-488 (BTX-488) and DAPI were imaged with a Zeiss Axioscope Spinning disk confocal microscope at 40× magnification with a water immersion lens coupled with Volocity software (version 6.3.0). AChR cluster size and number were quantified with ImageJ selection and counter tools respectively.

### 4.9. Statistical Analysis

All error bars on graphs represent the standard error of the mean (SEM). Unpaired t-tests were run to compare treated and untreated groups. Results from a treated group were normalized to the untreated group within each batch of an experiment. Statistical assessment was performed using GraphPad Prism 9 and MS Excel. Graphs were created with GraphPad Prism 9 and MS Excel.

## 5. Conclusions

Overall, this project demonstrates the relevance of investigating ALS hSKM as a therapeutic target. It also indicates the need for holistic approaches in ALS research (i.e., considering all factors involved) as opposed to the overemphasis on one tissue type. Lastly, this work demonstrates the utilization of BioMEMs technology as a valuable means of recapitulating complex human diseases like ALS and its outcomes for drug toxicological and efficacy screening.

## Data Availability

The data required to reproduce these findings cannot be shared at this time due to technical or time limitations.
